# The Influence of Tumor Microenvironment on ATG4D Gene Expression in Colorectal Cancer Patients

**DOI:** 10.1007/s12032-018-1220-6

**Published:** 2018-10-29

**Authors:** Justyna Gil, David Ramsey, Pawel Pawlowski, Elzbieta Szmida, Przemyslaw Leszczynski, Marek Bebenek, Maria M. Sasiadek

**Affiliations:** 1 Department of Genetics, Wroclaw Medical University, 50-368 Wroclaw, Poland; 2 Department of Operations Research, Wroclaw University of Technology, 50-372 Wroclaw, Poland; 3 Department of Biology and Medical Parasitology, Wroclaw Medical University, 50-345 Wroclaw, Poland; 4 First Department of Surgical Oncology, Lower Silesian Oncology Center, 53-413 Wroclaw, Poland

**Keywords:** Colorectal cancer, Autophagy, ATG, LC3, GABARAP, Relative expression

## Abstract

Despite great progress in research on the subject, the involvement of autophagy in colorectal cancer (CRC) pathogenesis (initiation, progression, metastasis) remains obscure and controversial. Autophagy is a catabolic process, fundamental to cell viability and connected with degradation/recycling of proteins and organelles. In this study, we aimed at investigating the relative expression level of mRNA via Real-Time PCR of 16 chosen genes belonging to Atg8 mammalian orthologs and their conjugation system, comprising *GABARAP, GABARAPL1, GABARAPL2, MAP1LC3A, MAP1LC3B, MAP1LC3C, ATG3, ATG7, ATG10, ATG4A, ATG4B, ATG4C, ATG4D*, and three genes encoding proteins building the multimeric ATG16L1 complex, namely *ATG5, ATG12*, and *ATG16L1*, in 73 colorectal tumors and paired adjacent normal colon mucosa. Our study demonstrated the relative downregulation of all examined genes in CRC tissues in comparison to adjacent noncancerous mucosa, with the highest rate of expression in both tumor and non-tumor tissues observed for *GAPARBPL2* and the lowest for *MAP1LC3C*. Moreover, in patients with advanced-stage tumors and high values of regional lymph nodes, statistically significant downregulation of *ATG4D* expression in adjacent normal cells was observed. Our study confirms the role of autophagy genes as cancer suppressors in colorectal carcinogenesis. Furthermore, in regard to the *ATG4D* gene, we observed the influence of tumor microenvironments on gene expression in adjacent colon mucosa.

## Introduction

Colorectal cancer (CRC) is among the leading causes of cancer-related death in the world (in fourth place among malignancies) [1]. In developed countries with high/very high human development indices (HDI), its incidence is higher. Although great progress has been made over the past decades in cancer prevention and identification as well as in anticancer therapy, including personalized therapy, the survival rate of CRC patients is still unsatisfying. However, in countries with high/very high HDIs (e.g., the USA, New Zealand), mortality rates have been stabilizing or declining, whereas in medium- to high-HDI counties (Eastern Europe, Asia) both incident and mortality rates have rapidly increased [1]. An understanding of cancer pathogenesis, along with a search for new prognostic biomarkers, may influence improvement in prognoses and may also enable the discovery of new potential targets for anticancer therapies. Much recent research has indicated autophagy as a potential new biomarker in CRC development [2].

Maintenance of cellular homeostasis plays an extraordinarily significant role in the normal growth and development of living organisms. In eukaryotic cells, autophagy maintains intracellular balance through the bulk degradation and recycling of redundant or damaged cytoplasmic proteins, aggregates, and/or organelles [3, 4]. Moreover, autophagy is involved in other physiological processes such as cell development, tumor suppression, immune defense, and response to stress; thus its deregulation has been linked to many diseases, including cancer [5]. This highly conserved process is strictly coordinated by core autophagy proteins encoded by more than 30 AuTophaGy-related (*ATG*) genes. The first step in this mechanism is the formation of an autophagosome, beginning with the creation of a cup-shaped membrane structure also known as an isolation membrane or phagophore. During elongation, the concave part of a phagophore becomes the luminal side of an autophagosome inner vesicle [6]. The next step in autophagy leads to the elongation and maturation of autophagosomes which fuse to lysosomes; thus, their contents are degraded by acidic hydrolases [7]. In autophagosome elongation, the pivotal role is played by mammalian homologs of the yeast Atg8 (autophagy-related 8) protein family. These homologs comprise two subfamilies: the microtubule-associated protein 1 light chain 3 MAP1LC3 including LC3A, LC3B, and LC3C, and the gamma-aminobutyric acid receptor-associated protein GABARAP, composed of three members: (i) GABARAP, (ii) GABARAPL1/GEC1/ATG8L (GABARAP-like protein 1/guinea pig endometrial glandular epithelial cells-1/Atg8-like protein), and (iii) GABARAPL2/GATE-16 (GABARAP-like protein 2/Golgi-associated ATPase enhancer of 16 kDa) [8–10]. All of these proteins contain highly conserved sequences (from yeasts to mammals), suggesting their important role in living cells [11]. Moreover, proteins belonging to these two subfamilies contain a conserved C-terminal glycine which is vital in autophagy [12]. The MAP1LC3 family is involved in phagophore elongation, whereas the GABARAP family is required in the later stages of autophagosome formation [12]. Apart from autophagy, Atg8 orthologs mediate in various intracellular trafficking processes [11]. The expression of Atg8 homolog proteins varies, depending on tissue type; e.g., LC3C is expressed mainly in the lungs, GABARAPL1 in the nervous system, GABARAPL2 in the brain, and GABARAP in the endocrine glands [11, 12].

Two ubiquitin-like conjugation systems are involved in the processing of Atg8 family members. One involves ATG7 (an E1-like enzyme) and ATG10, the other ATG3 (E2-conjugating enzyme) and ATG7; both mediate in the conjugation of the ATG16L1 complex (consisting of ATG5, ATG12, and ATG16). These enzymes, along with the Atg16L1 complex, are involved in the conjugation of phosphatidylethanolamine to yeast Atg8 human orthologs simultaneously with proteolytic maturation, which is dependent on ATG4 [13]. A certain role in the lipidation and delipidation of Atg8 homologs is played by the family of ATG4 cysteine proteases. Therefore, ATG4 activity is expected to be thoroughly regulated. At least, four human homologs are known to be involved in this process: (i) ATG4A (autophagin-2), (ii) ATG4B (autophagin-1), (iii) ATG4C (autophagin-3), and (iv) ATG4D (autophagin-4). Autophagins are responsible for the cleavage of various substrates of human Atg8 homologs [10, 14]. In most normal tissues, the expression of ATG4s is detected at low levels, with the highest level of expression of ATG4 proteins being observed in skeletal muscles as well as in the brain, heart, liver, pancreas, and testes [14]. Increased expression of ATG4 proteins has been shown to be connected with both the progression and suppression of tumor development as well as with cancer therapy resistance. Thus, these proteins may play a role as a potential/novel target in cancer therapy [14].

Therefore, in this study we aimed to investigate the mRNA expression of genes encoding pivotal proteins engaged in autophagosome elongation, such as *GABARAP, GABARAPL1, GABARAPL2, MAP1LC3A, MAP1LC3B, MAP1LC3*C, *ATG3, ATG4A, ATG4B, ATG4C, ATG4D, ATG5, ATG7, ATG10, ATG12*, and *ATG16L1*, in colorectal cancer tissue and paired adjacent normal colon mucosa.

## Materials and methods

The studied group was characterized in our previously published paper [15]. Briefly, samples of cancer tissue and paired normal adjacent mucosa obtained from 73 patients during surgery were collected and stored in accordance with recommended procedures. The clinical characteristics of the study group as well as the methodology of molecular and statistical analyses were precisely described previously [15]. Informed consent was obtained from each individual participant included in the study. The study design was accepted by the Wrocław Medical University Ethical Committee (approval number KB-822/2012).

In brief, mRNA was isolated from tumors as well as from noncancerous adjacent tissues and, following quality control, synthesis of cDNA was carried out. We employed the RealTime ready Assay with Universal ProbeLibrary (Roche) with *GABARAP* (ID 117150), *GABARAPL1* (ID 118062), *GABARAPL2* (ID 117687), *MAP1LC3A* (ID 144582), *MAP1LC3B* (ID 144005), *MAP1LC3C* (ID 144902), *ATG3* (ID 118148), *ATG4A* (ID 109561), *ATG4B* (ID 109269), *ATG4C* (ID 109546), *ATG4D* (ID 109054), *ATG5* (ID 125999), *ATG7* (ID 120541), *ATG10* (ID 138547), *ATG12* (ID 118103), and *ATG16L1* (ID113714) genes. Moreover, for normalization of Real-Time PCR results, three reference genes were applied [15].

The 2^−ΔΔCt^ method, Student’s *t* test, the Wilcoxon signed-rank test, Spearman’s correlation coefficient, and the Benjamini-Hochberg procedure were used for statistical analysis.

## Results

In both normal and tumor tissue, the highest levels of gene expression were observed for *GABARAPL2*, followed by *MAP1LC3B* and *MAP1LC3A* (see Table 1), the lowest for *MAP1LC3C* (see Table 1).

**Table 1:** Ranking of genes according to their absolute expression

Gene	*R*_N_	Δ_N_	95% CI	R_T_	Δ_T_	95% CI
*GABARAPL2*	1	2.3859	(2.2342, 2.5376)	1	3.2986	(3.1267, 3.4705)
*MAP1LC3B*	2	3.0610	(2.8849, 3.2371)	2	3.7988	(3.6073, 3.9903)
*MAP1LC3A*	3	4.1686	(3.9522, 4.3850)	3	4.5209	(4.3016, 4.7402)
*ATG12*	4	4.2312	(3.9492, 4.5132)	4	4.8904	(4.5544, 5.1228)
*ATG3*	5	4.6081	(4.5220, 4.6942)	5	4.8905	(4.7625, 5.0183)
*ATG5*	6	4.8188	(4.7017, 4.9359)	6	5.2499	(5.1097, 5.3901)
*ATG4D*	7	5.8584	(5.6341, 6.0827)	7	6.0616	(5.8688, 6.2544)
*ATG4A*	8	5.9106	(5.7112, 6.1100)	8	6.2979	(6.0823, 6.5135)
*ATG4C*	9	5.9419	(5.5782, 6.3056]	10	6.5358	(6.3620, 6.7096)
*ATG7*	10	6.3002	(6.1761, 6.4333)	9	6.3739	(6.2110, 6.5368)
*GABARAPL1*	11	6.3489	(6.0533, 6.6445)	12	7.5241	(7.2348, 7.8134)
*GABARAP*	12	6.9673	(6.7999, 7.1347)	13	7.7140	(7.4961, 7.9319)
*ATG4B*	13	7.0813	(6.8649, 7.2977]	11	7.3425	(7.1191 7.5659)
*ATG10*	14	7.5374	(7.3685, 7.7063)	14	8.4026	(8.1960, 8.6092)
*ATG16L1*	15	9.0196	(8.6257, 9.4135)	15	9.9371	(9.6926, 10.1816)
*MAP1LC3C*	16	9.9120	(9.4596, 10.3644)	16	10.8150	(10.0179, 11.6121)

R_N_ and R_T_ show the rankings for healthy and tumor cells, respectively. Similarly, Δ_N_ and Δ_T_ are the delta scores for healthy and tumor cells, respectively

For the ranking of genes according to the mean relative mRNA expression in all downregulated genes, see Table 2; for the ranking according to statistical significance, see Table 3.

**Table 2:** Ranking of genes according to mean relative fall in expression

Pos.	Gene	ΔΔ*CT*	95% CI	2^− ΔΔ*CT*^	95% CI
1	*GABARAPL1*	− 1.1751	(− 1.5688, − 0.7815)	2.2581	(1.7189, 2.9666)
2	*ATG16L1*	− 0.9175	(− 1.3111, − 0.5239)	1.8888	(1.4379, 2.4813)
3	*GABARAPL2*	− 0.9128	(− 1.1513, − 0.6742)	1.8827	(1.5957, 2.2211)
4	*MAP1LC3C*	− 0.9029	(− 1.6795, − 0.1264)	1.8698	(1.0916, 3.2032)
5	*ATG10*	− 0.8651	(− 1.1210, − 0.6092)	1.8215	(1.5254, 2.1750)
6	*GABARAP*	− 0.7467	(− 0.9653, − 0.5281)	1.6780	(1.4420, 1.9524)
7	*MAP1LC3B*	− 0.7378	(− 0.9945, − 0.4810)	1.6676	(1.3957, 1.9924)
8	*ATG12*	− 0.6074	(− 0.9147, − 0.3001)	1.5235	(1.2312, 1.8852)
9	*ATG4C*	− 0.5939	(− 1.0017, − 0.1861)	1.5093	(1.1377, 2.0024)
10	*ATG5*	− 0.4311	(− 0.5961, − 0.2660)	1.3483	(1.2025, 1.5116)
11	*ATG4A*	− 0.3873	(− 0.6409, − 0.1338)	1.3079	(1.0972, 1.5593)
12	*MAP1LC3A*	− 0.3523	(− 0.6398, − 0.0648)	1.2766	(1.0459, 1.5581)
13	*ATG3*	− 0.2823	(− 0.4308, − 0.1337)	1.2161	(1.0971, 1.3480)
14	*ATG4B*	− 0.2612	(− 0.5318, 0.0094)	1.1985	(0.9935, 1.4457)
15	*ATG4D*	− 0.1932	(− 0.4720, 0.0856)	1.1433	(0.9424, 1.3870)
16	*ATG7*	− 0.0737	(− 0.2671, 0.1197)	1.0524	(0.9204, 1.2034)

**Table 3:** Ranking of genes according to the significance of relative change in expression

Pos.	Gene	*p* value	Expression in Tumor
1	*GABARAPL2*	9.2408 × 10^‒10^	Lower
2	*ATG10*	1.2169 × 10^‒9^	Lower
3	*GABARAPL1*	5.5105 × 10^‒8^	Lower
4	*GABARAP*	7.0458 × 10^‒8^	Lower
5	*ATG5*	1.9192 × 10^‒7^	Lower
6	*MAP1LC3B*	6.2283 × 10^‒7^	Lower
7	*ATG16L1*	3.2873 × 10^‒6^	Lower
8	*ATG12*	0.0001977	Lower
9	*ATG3*	0.0003003	Lower
10	*ATG4A*	0.002327	Lower
11	*ATG4C*	0.003696	Lower
12	*MAP1LC3A*	0.01457	Lower
13	*MAP1LC3C*	0.03195	Lower
14	*ATG4B*	0.05435	No significant change
15	*ATG4D*	0.1673	No significant change
16	*ATG7*	0.4473	No significant change

When the Benjamini-Hochberg procedure for multiple testing was applied, each of the 13 significant differences explained above remained significant (tests used: Student’s *t* test and Wilcoxon rank test).

### Associations with expression levels in tumor tissue

#### Age

The expression levels of *ATG4B* and *MAP1LC3A* were negatively correlated with age (older people had higher scores on average) (Spearman’s correlation coefficient *R* = 0.294, *p* = 0.012, and *R* = 0.264, *p* = 0.024, respectively). Neither of these correlations was significant when the Benjamini-Hochberg procedure was applied.

#### Sex

The expression levels of *ATG7* and *GABARAPL1* were higher among males (lower mean scores): *p* = 0.006 and *p* = 0.044, respectively (see Table 4). These differences were not significant when the Benjamini-Hochberg procedure was applied.

**Table 4:** Expression of *ATG7* and *GABARAPL1* in tumor samples according to patients’ sex

Sex	*ATG7*	*GABARAPL1*
Female		
Mean	6.60244085	7.82499514
*N*	36	36
Standard deviation	0.663860195	1.268416464
Male		
Mean	6.15161525	7.23126054
*N*	37	37
Standard deviation	0.690844545	1.199491078
Total		
Mean	6.37394021	7.52406116
*N*	73	73
Standard deviation	0.710193119	1.261277719

#### Metastasis (M)

The expression level of *ATG3* was lower when M = 1 (*p* = 0.008) (see Table 5). These differences were not significant when the Benjamini-Hochberg procedure was applied.

**Table 5:** Expression of *ATG3* with regard to metastasis

M	*ATG3*
0	
Mean	4.84813801
*N*	65
Standard deviation	0.558581575
1	
Mean	5.34750717
*N*	7
Standard deviation	0.326935149
Total	
Mean	4.89668779
*N*	72
Standard deviation	0.558998761

#### Primary tumor (T)

T was positively correlated with expression levels at *GABARAP1* (high T was correlated with low scores) (Spearman’s correlation coefficient *R* = − 0.250; *p* = 0.033).

#### TNM advancement

The level of advancement was negatively associated with expression at *MAPILC3A* (Spearman’s correlation coefficient *R* = 0.350; *p* = 0.002).

#### Regional lymph nodes (*N*)

High values of *N* (2 and 3) were associated with low levels of expression of *MAP1LC3A* in tumor cells (*p* = 0.012, analysis of variance, see Table 6).

**Table 6:** Expression of *MAP1LC3A* in tumor cells in the context of regional lymph nodes

*N* (nodes)	Mean	*n*	Standard deviation
0	4.27632598	8	0.604827086
1	4.29572818	40	0.857568475
2	4.77995641	20	0.965130494
3	6.27228457	3	1.193241333
Total	4.51346080	71	0.962399452

### Associations with expression levels in “normal” cells (adjacent normal tissue)

#### Age

Expression of *GABARAP* (Spearman’s correlation coefficient *R* = − 0.256; *p* = 0.029) and of *ATG7* (Spearman’s correlation coefficient *R* = − 0.241; *p* = 0.040) was positively correlated with age (older people had, on average, lower scores). None of these correlations were significant when the Benjamini-Hochberg procedure was applied.

#### Sex, location, M

There was no significant association between sex, location of the tumor, the value of M, or the difference in expression levels between healthy and tumor cells of any gene.

#### Primary tumor (T)

T was negatively correlated with expression in healthy cells in the two following genes: *ATG4B* (Spearman’s correlation coefficient *R* = 0.279; *p* = 0.018) and *ATG5* (Spearman’s correlation coefficient *R* = 0.255; *p* = 0.029).

#### TNM advancement

Low levels of expression of *ATG4D* were associated with stage III (analysis of variance, *p* = 0.006) (see Table 7).

**Table 7:** Expression of *ATG4D* in adjacent normal tissue

TNM classification	Mean	*N*	Standard deviation
I	4.24223198	3	0.977804358
II	5.32752781	6	0.631598190
IIIA	6.22694475	4	0.905514822
IIIB	6.09223163	40	0.980801674
IIIC	5.90147052	13	0.773275607
IV	5.48368794	7	0.892834547
All	5.86840876	73	0.977842805

#### Regional lymph nodes (*N*)

High values of *N* (2 and 3) are associated with low levels of expression of *ATG4D* in healthy cells (*p* = 0.027, analysis of variance). Nx was excluded from the analysis (see Table 8).

**Table 8:** Mean expression of the *ATG4D* gene in normal tissue

*N*	Mean	*N*	Standard deviation
0	5.93913209	8	1.114719687
1	5.96140806	40	0.876880964
2	6.32719456	20	0.651688059
3	6.18172253	3	0.746205113
All	6.07124561	71	0.843783978

### Associations with differences in expression levels between tumor and healthy cells

Age was positively correlated with the fall in expression levels between healthy and tumor cells of the three following genes: *ATG4B* (Spearman’s correlation coefficient *R* = 0.352; *p* = 0.002), and *MAP1LC3A* (Spearman’s correlation coefficient *R* = 0.279; *p* = 0.017). The association between age and the fall in expression of *ATG4B* remained significant when the Benjamini-Hochberg procedure for multiple testing was applied.

#### Regional lymph nodes (*N*)

*N* = 1 is associated with a lack of difference in levels of expression of *ATG4D* between healthy and tumor cells. Other values of *N* are associated with lower levels of expression in tumor cells than in healthy cells (*p* = 0.038, analysis of variance) (see Table 9). Nx was excluded from the analysis.

**Table 9:** Result of differential expression of the *ATG4D* gene between tumor and normal cells

*N*	Mean	*N*	Standard deviation
0	0.9379	8	1.05433
1	‒0.1703	40	1.28877
2	0.5695	20	0.91082
3	0.4234	3	0.92888
Total	0.1881	71	1.21159

## Discussion

Altered expression (either under- or over-) of autophagy genes may be an important factor in the development and progression (metastases formation) of various human malignancies [16, 17]. Despite the immense efforts exerted in this area, the role of autophagy in carcinogenesis remains controversial. Although dysregulation of the key autophagy proteins Beclin 1 and LC3 have been studied in a wide variety of tumors, the role of these proteins in colorectal cancer is ambiguous. The first study on LC3 expression in gastrointestinal cancers (esophageal, gastric, and colorectal) employing immunohistochemistry was performed by Yoshioka et al., who showed that, in all examined cancer types, LC3 expression was significantly elevated in comparison to normal adjacent tissue. Interestingly, no significant association was found between clinicopathological parameters and LC3 expression [18]. Subsequent studies have also reported upregulation of LC3 expression in CRC tissues as well as in CRC cell lines [19–21]. However, in most cases the specificity of the relevant LC3 antibodies has not been examined. Moreover, other studies, including ours, have produced contradictory results [15, 22].

In this study, we found relative downregulation of all 16 examined genes. Among genes with extremely low levels of expression were *MAP1LC3A, MAP1LC3B*, and *MAP1LC3C*. Intriguingly, Bai et al. showed that *LC3Av1* was commonly silenced at the transcriptional level in numerous cancer cell lines due to epigenetic changes. Notably, other research revealed that *BNIP3*, another gene engaged in the regulation of autophagy, is also silenced in CRC cell lines by the methylation of CpG islands as well as by histone deacetylation [23]. Hence, these observations implied epigenetic modifications as an important factor modifying autophagy expression in cancer cells.

Yang et al. demonstrated underexpression of Beclin 1 and LC3B in the central area of a tumor compared to the adjacent noncancerous mucosa. Additionally, expression of the analyzed proteins has been shown to be strongly associated with patients’ survival. Thus, it has been hypothesized that the expression patterns of the autophagic proteins in the adjacent noncancerous “normal” tissues may also serve, albeit to a lesser degree, as predictive markers of the clinical outcomes of diseases. This is possible due to the strong affect exerted by the tumor’s microenvironment on autophagic flux in the adjacent mucosa, which may influence the promotion of tumor proliferation [24]. However, we found no association between normal cells and expression of *MAP1LC3A, MAP1LC3B*, or *MAP1LC3C*.

Nevertheless, in regard to the specific tumor microenvironment, we found statistically significant downregulation of *ATG4D* expression in adjacent normal cells in patients with high tumor stages and high values for regional lymph nodes. *ATG4D* encodes a protein belonging to the ATG4 mammalian family (the class of four cysteine proteases, ATG4A‒D) presenting endopeptidase activity, vital for later stages of autophagosome maturation and fusion with lysosomes [25]. ATG4D, one of the above-mentioned four proteases, contains a domain (DEVD) cleaved by caspase-3. The truncated form of ATG4D, compared to its full-length form, shows increased priming and delipidating activity against *GABARAPL1* [26]. It has been shown that silencing of ATG4D expression sensitizes HeLa cells and lead to starvation-induced cell death, indicating that ATG4D-dependent autophagy administers to cellular survival [9, 26]. Furthermore, ATG4D has been linked to apoptosis pathways, as its overexpression may induce this process. In cells treated with hydrogen peroxide, cleaved ATG4D is recruited to dysfunctional mitochondria.

In conclusion, the expression of ATG4D and other ATG4 isoforms may regulate the post-translational activation of the LC3/GABARAP family proteins [9, 26, 27].

Our analysis indicates that, in patients with advanced stages of CRCs, the expression level of this vital enzyme is lower in normal tissues. Therefore, in this case, we probably observed a “field effect” and thus hypothesized that ATG4D plays a very important role in CRC development.

Interestingly, our study demonstrated that, in tumor cells, the low level of expression of *ATG4B* as well as of the previously mentioned *MAP1LC3A* was associated with age. This observation suggests that decreasing levels of enzyme ATG4B, which mediates the conversion of pro-LC3 isoforms to the LC3-I form (a substrate for subsequent lipidation to LC3-II), may be connected with both cancer development and the aging of patients.

ATG4B is involved in post-translational processing of LC3; however, our results indicate that depleted expression of *ATG4B* mRNA may trigger the reduced expression of genes encoding post-translational target for this gene. Because of the critical role of ATG4B in the autophagy process, an inhibitor of this protease has been proposed as a promising tool for monitoring the treatment not only of cancers but also of other diseases [28]. Nonetheless, to date, no data confirming the prognostic value of any ATG4 family member in cancers have been published [8].

Our research has shown that *GABARAPL2* is the most prominently expressed gene in both normal and tumor tissue. The differing expressions of highly homological proteins from the GABARAP family are observed in both developing and adult tissues. These proteins play an important role in various cellular processes, including receptor transport, cell proliferation, and autophagy [29]. *GABARAPL1* is expressed in numerous tissues, including the brain, heart, skeletal muscles, kidney, ovary, and blood lymphocytes. It has been shown that estrogens may influence the expression of *GABARAPL1* and hence may be involved in a variety of estrogen-sensitive diseases such as breast cancer [12]. The expression of the GABARAP family has been studied, *inter alia*, in breast cancer (BC). In cancer tissue, only the *GABARAPL1* mRNA level was shown to be significantly reduced, whereas those of *GABARAP* and *GABARAPL2* were not. The downregulation of *GABARAPL1* in BC has been linked to gene silencing via promoter hypermethylation and histone H3 deacetylation. Hence, it has been proposed that epigenetic inhibitors could be used along with classical chemotherapeutics in BC therapy [29]. Notably, in our study we found the *GABARAPL1* gene to be the most silenced.

Hitherto, only one research project has examined the prognostic significance of the GABARAP protein in colorectal cancer, thus showing that high levels of expression of this protein were connected to poor differentiation and to shortened overall survival in CRC patients [30].

As of now, there is very little information on the mRNA levels in genes encoding ATG16L complex in colorectal cancer. We found variable mRNA expression levels in genes encoding proteins creating this complex, such as high *ATG12*, median *ATG5*, and low *ATG16L1. ATG5* and *ATG12* create an ubiquitin-like conjugate which interacts with *ATG16L* in a noncovalent manner and creates a multimeric complex crucial for elongation of the pre-autophagosomal membrane [31]. Experimental studies on mice models have shown that heterozygous deletion of *ATG5* is involved in intestinal tumor growth and sensitizes tumor cells to chemotherapy [32]. Moreover, it has been demonstrated that *ATG5* silencing by specific short interfering RNAs may strengthen the efficacy of chemotherapeutic agents during combination therapy. Interestingly, interferon-gamma has revealed a strong inhibition effect on adenomas of Apc^Min^/+ATG5+/−mice without obvious side effects. Therefore, a new anticancer treatment strategy based on interferon-gamma and ATG5-targeted inhibition has been proposed [32]. Strong downregulation of ATG5 expression in CRC samples as well as in colorectal cell lines was observed as used both Western blot analysis and IHC assay [33]. Moreover, a reduced level of the conjugated form ATG5‒ATG12 in CRC samples in comparison to normal mucosa has been observed (data not shown) [33]. Our observations may indicate that ATG16L1 plays a regulatory role in the ATG16L complex because of its very limited expression in both normal and cancer tissue.

## Conclusions

To conclude, in our research, we observed relative mRNA downregulation of all examined genes in colorectal cancer tissues. In normal cells of patients with advanced cancer stages, we found statistically significant downregulation of the *ATG4D* gene. A mean relative decrease in expression was most obvious for *GABARAPL1*. These findings may indicate the genes in question as potential biomarkers as well as putative targets in CRC diagnosis and therapy. However, our limited cohort and lack of biological/functional analyses indicate that further characterization is required.

## References

[1] Arnold M, Sierra MS, Laversanne M, Soerjomataram I, Jemal A, Bray F (2017). Global patterns and trends in colorectal cancer incidence and mortality. Gut.

[2] Gil J, Pesz KA, Sąsiadek MM (2016). May autophagy be a novel biomarker and antitumor target in colorectal cancer?. Biomark Med.

[3] Klionsky DJ (2007). Autophagy: from phenomenology to molecular understanding in less than a decade. Nat Rev Mol Cell Biol.

[4] Behrends C, Sowa ME, Gygi SP, Harper JW (2010). Network organization of the human autophagy system. Nature.

[5] Mizushima N, Levine B (2010). Autophagy in mammalian development and differentiation. Nat Cell Biol.

[6] Yu Z-Q, Ni T, Hong B (2012). Dual roles of Atg8-PE deconjugation by Atg4 in autophagy. Autophagy.

[7] Klionsky DJ, Codogno P, Cuervo AM (2010). A comprehensive glossary of autophagy-related molecules and processes. Autophagy.

[8] Bortnik S, Gorski SM (2017). Clinical applications of autophagy proteins in cancer: from potential targets to biomarkers. Int J Mol Sci.

[9] Schaaf MBE, Keulers TG, Vooijs MA, Rouschop KMA (2016). LC3/GABARAP family proteins: autophagy-(un)related functions. FASEB J.

[10] Wild P, McEwan DG, Dikic I (2014). The LC3 interactome at a glance. J Cell Sci.

[11] Shpilka T, Weidberg H, Pietrokovski S, Elazar Z (2011). Atg8: an autophagy-related ubiquitin-like protein family. Genome Biol.

[12] Le Grand JN, Chakrama FZ, Seguin-Py S (2011). GABARAPL1 (GEC1): original or copycat?. Autophagy.

[13] Galluzzi L, Baehrecke EH, Ballabio A (2017). Molecular definitions of autophagy and related processes. EMBO J.

[14] Zhang L, Li J, Ouyang L, Liu B, Cheng Y (2016). Unraveling the roles of Atg4 proteases from autophagy modulation to targeted cancer therapy. Cancer Lett.

[15] Gil J, Ramsey D, Szmida E (2017). The BAX gene as a candidate for negative autophagy-related genes regulator on mRNA levels in colorectal cancer. Med Oncol.

[16] Saha S, Panigrahi DP, Patil S, Bhutia SK (2018). Autophagy in health and disease: a comprehensive review. Biomed Pharmacother.

[17] Dower CM, Wills CA, Frisch SM, Wang H-G (2018). Mechanisms and context underlying the role of autophagy in cancer metastasis. Autophagy.

[18] Yoshioka A, Miyata H, Doki Y (2008). LC3, an autophagosome marker, is highly expressed in gastrointestinal cancers. Int J Oncol.

[19] Chen Z, Li Y, Zhang C (2013). Downregulation of Beclin1 and impairment of autophagy in a small population of colorectal cancer. Dig Dis Sci.

[20] Guo G-F, Jiang W-Q, Zhang B (2011). Autophagy-related proteins Beclin-1 and LC3 predict cetuximab efficacy in advanced colorectal cancer. World J Gastroenterol.

[21] Choi JH, Cho Y-S, Ko YH, Hong SU, Park JH, Lee MA (2014). Absence of autophagy-related proteins expression is associated with poor prognosis in patients with colorectal adenocarcinoma. Gastroenterol Res Pract.

[22] Chang Y-T, Tseng H-C, Huang C-C, Chen Y-P, Chiang H-C, Chou F-P (2011). Relative down-regulation of apoptosis and autophagy genes in colorectal cancer. Eur J Clin Invest.

[23] Murai M, Toyota M, Suzuki H (2005). Aberrant methylation and silencing of the BNIP3 gene in colorectal and gastric cancer. Clin Cancer Res.

[24] Yang M, Zhao H, Guo L (2015). Autophagy-based survival prognosis in human colorectal carcinoma. Oncotarget.

[25] El Andaloussi A, Habib S, Soylemes G (2017). Defective expression of ATG4D abrogates autophagy and promotes growth in human uterine fibroids. Cell death Discov.

[26] Betin VMS, Lane JD (2009). Caspase cleavage of Atg4D stimulates GABARAP-L1 processing and triggers mitochondrial targeting and apoptosis. J Cell Sci.

[27] Costa JR, Prak K, Aldous S, Gewinner CA, Ketteler R (2016). Autophagy gene expression profiling identifies a defective microtubule-associated protein light chain 3A mutant in cancer. Oncotarget.

[28] Nguyen TG, Honson NS, Arns S (2014). Development of Fluorescent Substrates and Assays for the Key Autophagy-Related Cysteine Protease Enzyme, ATG4B. Assay Drug Dev Technol.

[29] Hervouet E, Claude-Taupin A, Gauthier T (2015). The autophagy GABARAPL1 gene is epigenetically regulated in breast cancer models. BMC Cancer.

[30] Miao Y, Zhang Y, Chen Y, Chen L, Wang F. GABARAP is overexpressed in colorectal carcinoma and correlates with shortened patient survival. Hepatogastroenterology. 57:257–61. 20583424

[31] Glick D, Barth S, Macleod KF (2010). Autophagy: cellular and molecular mechanisms. J Pathol.

[32] Wang L, Wang Y, Lu Y, Zhang Q, Qu X (2015). Heterozygous deletion of ATG5 in *Apc*^Min/+^ mice promotes intestinal adenoma growth and enhances the antitumor efficacy of interferon-gamma. Cancer Biol Ther.

[33] Cho D-H, Jo YK, Kim SC, Park IJ, Kim JC (2012). Down-regulated expression of ATG5 in colorectal cancer. Anticancer Res.

